# The Electronic Medical Record and Fraudulent Billing

**DOI:** 10.1016/j.mcpdig.2023.03.009

**Published:** 2023-05-01

**Authors:** Edward P. Hoffer

**Affiliations:** Harvard Medical School, Boston, MA

Although electronic medical records (EMRs) offer many advantages, they have their drawbacks, which must be addressed by the medical profession.

Some years ago, I referred a patient with persistent sciatic pain to a neurosurgeon to discuss possible surgical intervention. Soon after the visit, I got a very detailed note back. The note described a thorough history, including social and family history, past medical history, and a detailed negative review of systems. There was also a comprehensive physical examination described that included normal funduscopic and rectal examination results. When I next saw the patient, I asked him to describe his visit with “Dr. X.” The patient replied “he had me sitting on the exam table in a johnny. He lifted each of my legs, tapped on my knees, and then reviewed the CT exam.” Almost none of the extensive history and physical examinations described in the visit note had occurred.

That was neither the first nor the last time I received such correspondence. EMRs make it much too easy to populate a note with precanned normal items from history and physical examinations using just a few clicks. Is this fraud? I believe it is, aided and abetted by EMRs.

Evaluation and management Current Procedural Terminology codes have always been difficult to assign with complete accuracy; however, pre-EMRs were harder to deliberately falsify.^1^

Another recent phenomenon that has accompanied the rapid growth of the use of EMRs is “note bloat.” An oncologist to whom I had referred many patients sent me 4- and 5-page notes after each patient encounter. Ninety percent or more of each note consisted of “cut-and-paste” from previously reported scans as well as pathology and laboratory reports. It took considerable time parsing these tracts to find out what had happened at the actual visit.

The obvious reason for this behavior is “upcoding,” using a voluminous note, padded with details that were either not elicited at the visit or previously reported and irrelevant to the service performed, to justify a higher billing code. Upcoding has been with us for a while but is becoming steadily more prevalent.^2^

It has been documented that emergency physicians have shifted their billing codes higher since the introduction of EMRs.^3^ Are patients truly sicker than they were a few years ago or have EMRs been manipulated to give the appearance of a more thorough examination during visits?

Although fraudulent billing has always existed, it has become more flagrant in the EMR era. Physicians hand writing their notes were much less likely to describe things they had not done. The temptation to use a few clicks appears to be too great for some to resist. The medical profession must take responsibility to stamp out this egregious practice, or a solution will be imposed on us.^4^

## Potential Competing Interest

The author reports no competing interests.
